# Approaching the Communication Constraints of Ethereum-Based Decentralized Applications

**DOI:** 10.3390/s19112647

**Published:** 2019-06-11

**Authors:** Matevž Pustišek, Anton Umek, Andrej Kos

**Affiliations:** Faculty of Electrical Engineering, University of Ljubljana, Tržaška 25, SI-1000 Ljubljana, Slovenia; anton.umek@fe.uni-lj.si (A.U.); andrej.kos@fe.uni-lj.si (A.K.)

**Keywords:** architecture, blockchain, communication constraints, decentralized application, Ethereum, Internet of things

## Abstract

Those working on Blockchain technologies have described several new innovative directions and novel services in the Internet of things (IoT), including decentralized trust, trusted and verifiable execution of smart contracts, and machine-to-machine communications and automation that reach beyond the mere exchange of data. However, applying blockchain principles in the IoT is a challenge due to the constraints of the end devices. Because of fierce cost pressure, the hardware resources in these devices are usually reduced to the minimum necessary for operation. To achieve the high coverage needed, low bitrate mobile or wireless technologies are frequently applied, so the communication is often constrained, too. These constraints make the implementation of blockchain nodes for IoT as standalone end-devices impractical or even impossible. We therefore investigated possible design approaches to decentralized applications based on the Ethereum blockchain for the IoT. We proposed and evaluated three application architectures differing in communication, computation, storage, and security requirements. In a pilot setup we measured and analyzed the data traffic needed to run the blockchain clients and their applications. We found out that with the appropriate designs and the remote server architecture we can strongly reduce the storage and communication requirements imposed on devices, with predictable security implications. Periodic device traffic is reduced to 2400 B/s (HTTP) and 170 B/s (Websocket) from about 18 kB/s in the standalone-device full client architecture. A notification about a captured blockchain event and the corresponding verification resulted in about 2000 B of data. A transaction sent from the application to the client resulted in an about 500 B (HTTP) and 300 B message (Websocket). The key store location, which affects the serialization of a transaction, only had a small influence on the transaction-related data. Raw transaction messages were 45 B larger than when passing the JSON transaction objects. These findings provide directions for fog/cloud IoT application designers to avoid unrealistic expectations imposed upon their IoT devices and blockchain technologies, and enable them to select the appropriate system design according to the intended use case and system constraints. However, for very low bit-rate communication networks, new communication protocols for device to blockchain-client need to be considered.

## 1. Introduction

The Internet o things (IoT) [1] is a well-established concept referring to numerous interconnected things along with corresponding cloud or fog/edge-based applications. It is revolutionizing the Internet and is being deployed in a variety of application domains. The distributed ledgers on the other hand—which are currently mostly implemented with blockchain technologies (BC)—are still emerging [2]. Nevertheless, they are likely to disrupt the field of ICT systems, services, and applications just as strongly as the IoT has in the past. There have already been initial attempts to jointly use the IoT and distributed ledgers [3,4,5,6,7,8,9,10]. These attempts tend to study the feasibility of such application development approaches, provide proofs of concepts (PoC), explore possible use cases, and highlight future business opportunities. Despite the broken illusions about cryptocurrencies in 2018, the technological development in blockchain technologies continues. It started focusing even more on non-monetary applications, including the IoT. This includes seeking performance in BC networks, investigating the role of artificial intelligence for the blockchain, and developing tools, middleware, and service frameworks for real-world business.

The scope of the existing BC systems is divergent in terms of technological features, as well as in their acceptance among the user- and developer communities. In the decade since their first introduction, BC research and development focused on enabling technologies and first BC-based decentralized applications. With the first examples of BC-based IoT solution deployments, certain inefficiencies in current BC designs started appearing. Micropayments for example have become almost unrealistic in the Bitcoin (or Ethereum) network due to high transaction fees and long transaction confirmation times. The scalability and performance needed for the IoT (expected billions of devices) is often limited due to the size of the blockchain and limited transaction rates and excessive latency. The existing BC protocols, e.g., Bitcoin [11] and Ethereum [12], endeavor to face some of these inefficiencies with functional extensions, such as state channels [13,14], sharding [15], and oracles [16]. In parallel, new ledger protocols are being developed, e.g., the Hyperledger Fabric (HLF) [17], NEO [18], IOTA [19], Cardano [20] or Stellar [21] with some of the IoT requirements built-in from scratch. Both developments—the IoT and the BC—are naturally seeking to be combined in common solutions, which thus provide an immense space for application development and use. However, the right approach and the selection of appropriate technologies are far from being straightforward. Beside the differences in technological features, issues like the scalability, transaction costs, deployment in constrained IoT devices must be considered. The same holds for the acceptance of a particular blockchain technology among user and developer communities. The selection can crucially depend on the details of an intended use case, too. Despite a variety of existing and emerging BC technologies, Ethereum is by far the most popular platform for IoT BC applications. Among eleven cases from different application domains presented [22], seven are based on Ethereum and the remaining four are multiplatform (i.e., including the Ethereum). 

Interestingly, very little scientific research can be found on actual system and communication requirements to run IoT devices with full blockchain support. For Ethereum even the developers’ documentation and installation instructions do not clearly state the necessary allocation of resources to run a full blockchain client successfully. Various user reports [23,24,25] indicate that at least 2-4 GB RAM and extensive swap sizes are needed to run the client as a full node. For Bitcoin the instructions state just that one needs “a desktop or laptop hardware running recent versions of Windows, Mac OS X, or Linux” and 2 GB RAM [26] for the full client. The storage requirements are determined by the size of the full blockchain, which in May 2019 was about 204 GB in Bitcoin [27], 130 GB in Ethereum with fast sync option applied [28], and 225 GB for a full node [29]. Both are constantly increasing at about 0.1-0.5 GB per day and resulting in approximately 2‑5 kB/s of constant communication traffic. In IOTA the requirements are not explicitly defined either, but seem to be comparable to the ones in Ethereum. Block data snapshots are applied in IOTA, which is similar to the pruning concept in blockchain. The storage requirements in IOTA are therefore lower, but can still reach several dozens of GB per node. Despite lacking the precise numbers for the requirements, these figures exceed the capacities of embedded IoT devices. 

We therefore face a research and engineering challenge, namely how to bring the blockchain capabilities to the IoT devices that can be constrained in CPU, RAM, storage, communication bandwidth, and energy consumption, and the like. Not all these constraints are necessarily present in every element of an IoT system. The second challenge refers to the security implications of the selected architectural approaches for applications of blockchain technologies for IoT. 

In this article we:

(1) Present the practical constraints from the perspective of end devices in the development of IoT applications based on the Ethereum blockchain as one of the viable and very popular platform for IoT BC applications.
(2) Elaborate and compare in terms of computation and communication constraints, as well as in terms of security, three architectural approaches for the design of IoT end-device applications based on the Ethereum BC.
(3) Analyze the results of communication traffic measurements in these architectures to clearly estimate the communication constraints.

Our research provides directions for IoT application designers to enable them to select the appropriate system design and avoiding the placement of unrealistic expectations on IoT devices and BC technologies. Their architectural approach can thus be shaped according to the intended use and the specifics of the planned IoT system.

In Section 2 we briefly present the related work and indicate possible use of decentralized BC applications in IoT. In Section 3 we outline the principles of distributed BC application development for the IoT based on the Ethereum. These principles are elaborated into three architectural approaches for BC enabled IoT devices—Section 4—which differ in communication and computation constraints, as well as in in their security implications. In our pilot installation we measured and analyzed the traffic in various architectures. These results are presented in Section 5 and discussed in Section 6. 

## 2. Related Work

There are not many successful use cases of IoT BC solutions with important and practical business impacts that also reach beyond a proof-of-concept (PoC) and incorporate more than just a limited number of devices. This is not surprising, as the application domain of IoT with blockchain is still in its infancy. Current activities are directed primarily towards the clarification of the role of the BC in the IoT, testing limitations in implementation, and exploring possible business opportunities. Nevertheless, interesting use cases have been presented, primarily in the domains of smart home, smart grid and electric charging, logistics, and IoT device management. 

The adoption of BC in IoT is analyzed in [30]. It highlights three significant challenges such as a high resource demand, long latency, and low scalability. It proposes an architecture that combines private and public BC, with simplified block management and cluster headers as gateway entities that provide public BC functionalities for other devices in the system. The same first author in [31] presents the optimization of the BC in the context of smart homes. They analyze security and privacy aspects, along with the overhead introduced by the BC, which remains low and manageable even for resource-constrained devices. Blockchain and IoT integration is further investigated in [32] and in [22].

In [33] they investigate the role of the BC in the mobile charging of electrical vehicles. The concept is presented generally and does not refer to any specific BC technology. The need for the dissemination of chain blocks at all charging stations is not clearly justified and it seems as if a traditional server-based solution could do the job, too. Nevertheless, they point out problems of running full BC clients on constrained nodes. They suggest light clients (Simplified Payment Verification, SPV) and a reduced number of full nodes acting as gateways (called Service Provider, SP).

In [34] they present a prototype of an end-to-end solution, based on an Ethereum BC-controlled IoT electric switch. They implemented the hardware and software for the device, along with the smart contract and the Ethereum compliant web applications for use and control of the system. The device was later upgraded to measure consumption, too. It thus acts as an independent BC node, reporting the measurement status into the chain. In [35] they investigate the support of BCs in various IoT platforms and in [36] they analyze the requirements of IoT devices for the Ethereum BC.

There are other examples of electricity-related use cases of BC technologies for IoT that rely on current public BC networks. In SGs the key challenges that are currently being addressed with the IoT and BC are smart meter reading, selling surplus energy in local microgrids, electric vehicle charging, and demand side management [6,37]. 

Other application domains present interesting cases of IoT blockchain applications [22], too. Logistics companies are investigating the role of the IoT and BC for product identification and tracking cargo shipments. The idea behind the SmartCargo [7] is that the shipping process should be automated, secure, and transparent throughout the logistic process. Their solution is based on IoT and blockchain and, inter alia, gives access to trustworthy and live cargo tracking. In [4] a container tracking solution is presented that measures light, temperature, and other environmental parameters, and then secures this information in a blockchain. In [5] a similar approach is applied in the pharma supply chain. IoT device management is fundamental to other application domains because it includes access and storage of IoT data in BCs. In [38] this concept is proven in a smart-home scenario to manage home appliances and electricity consumption. A similar idea is elaborated in [39] for the management of vending machines. 

Authors in [8] address privacy risks and security concerns in IoT-based healthcare applications. They propose a framework with additional privacy and security properties in a blockchain for IoT, to provide secure management and analysis of healthcare-related big data.

In an envisaged on-demand insurance scenario the study [9] combines blockchain technology with IoT sensors installed in a vehicle. Their proposed system, which is an example of a decentralized blockchain IoT application, enables semi-automatic activation of car insurance coverage. 

IoT security, too, can be greatly supported by blockchain technologies. In [40] they elaborate various challenges in effectively implementing security for IoT devices, including resource limitations, device heterogeneity, interoperability of (security) protocols, and scalability and latency of BC networks. Another study [10] elaborates options for access management in IoT. It provides an architecture of a fully distributed access control system based on Ethereum blockchain technology for arbitrating roles and permissions in IoT.

### Ethereum as the Ledger Technology for the IoT

In [41] the authors provide a systematic overview of BC technologies and smart contracts for the IoT. They identify several issues that may come up when IoT makers experiment further with BCs, and have their IoT devices participate in a BC network. In this respect they point out the limited transaction throughput (compared to the traditional databases), privacy in the BC, the appropriate selection of BC miners to prevent transaction censoring, the limited legal enforceability of smart contracts, and smart contract validation and security. Rapid developments of blockchain protocols and practical deployments in proofs-of-concepts pointed out that some of the expectations [42] that in the past were placed on blockchain technology for the IoT cannot be taken for granted. Especially current major public BC networks, with their still-present scalability, delay, and cost issues, indicate the need for a clear understanding of new architectural options and required protocol enhancements. An insight into the expectations, actual position and possible remedies is given in Table 1.

The existing BC protocols try to cope with their limitations by making additions that more or less successfully patch the core BC protocols. The state channels, for example, in comparison to the current BC architectures, combine off- and on-chain transactions to contribute to additional scalability, privacy, and the reduction of confirmation delays. In Ethereum this approach is manifested in the Generalized state channels and µRaiden and Raiden networks [13], and in BTC in the Lightning network [14]. The Ethereum smart contracts cannot contact external URLs, which limits their integration with the “world outside of the chain”. This shortcoming could be outdone by oracles [16]. These serve as intermediaries, providing data feeds along with an authenticity proof to the blockchain from/to external software (e.g., web sites) or hardware entities. These add-ons have garnered some interest, but are not yet mature (e.g., strong mismatches between announced roadmaps and actual dates of delivery) and with little practical acceptance. This explains why IOTA took a different approach, where the ledger technology (and entire system around it) was designed for the IoT from the very beginning. 

In this article we elaborate upon the architectures of IoT devices and applications that are based on the Ethereum network. With this selection we in no way wish to single it out as the only appropriate ledger technology in this context. However, the Ethereum network has a proven record in supporting smart contracts, mature implementations of blockchain clients and programming libraries, a large application development community, industry support, and many successful examples of interesting decentralized applications (DApps) already developed. The Ethereum protocols can be implemented in private networks, too, and this can be another option for alleviating, e.g., scalability constraints or transaction costs. For these reasons it is always an option that has to be seriously considered as a viable technological candidate. 

The Ethereum protocol, which is being developed by The Ethereum Foundation, is specified in the Yellow paper [43]. New Ethereum transactions are formed in blocks that are validated by mining nodes. The miners use consensus algorithms for validation and they are rewarded for their work. The Ethereum nodes can participate in various public networks, as for example the mainnet or the test network Ropsten.

The key innovation in the Ethereum protocol compared to BTC is the support of smart contracts (SC). These are not some formal requirements or obligations, but can be more adequately explained as autonomous agents, whose behavior is determined by their contract code. This code is executed every time this account receives a message, which is a transaction addressed to it. To develop smart contracts and thus the decentralized applications, a computationally universal (i.e., Turing complete) language is provided. The fundamental smart contract language is the low-level bytecode language and the Ethereum network provides a virtual machine (i.e., Ethereum virtual machine, EVM) which executes such code. Several high(er) level languages are available for application development. The current flagship is Solidity [44]—a JavaScript like language—but other languages have been used in the past. Higher-level code is compiled to bytecode prior to execution in the EVM.

## 3. Decentralized Applications with Ethereum

**Figure 1:**
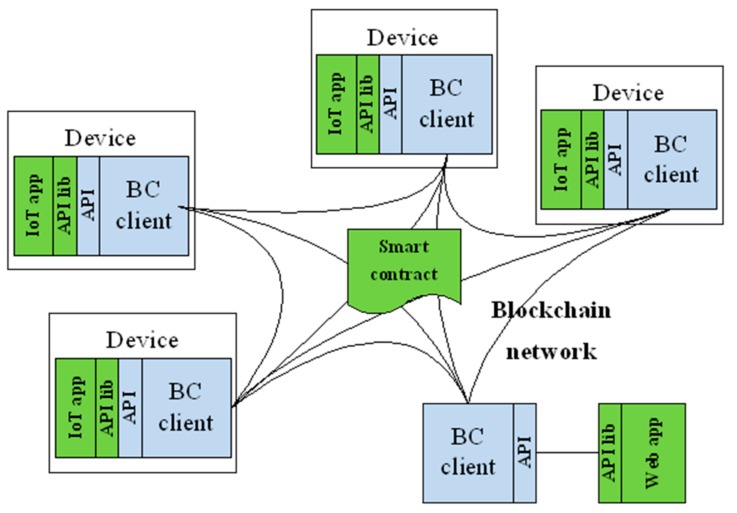
Ethereum’s decentralized application architecture for the IoT.

Distributed ledgers provide a trusted environment for transactions. In terms of application development for the IoT, two paradigms can be combined—IoT applications with BC support and on-chain logic. Both parts together comprise a decentralized application (DApp), which utilizes the blockchain. Depending on the intended use, both application parts can be combined into one solution, as depicted in Figure 1.

- On-chain application logic refers to smart contracts (i.e., chaincode in Hyperledger Fabric (HLF)), which are programs deployed and executed in the BC network. Executions of smart contracts are validated in BC. BC thus provides a decentralized and trusted virtual machine for smart contract executions. The on-chain logic is not absolutely required for the IoT.
- IoT applications with BC support are web, mobile and embedded applications, which use the BC via client APIs that are made available by the BC clients. These parts of decentralized applications are required for user interfaces and for IoT devices to utilize the BC.

### 3.1. On-Chain Application Logic

The decentralized environment for trusted transactions, which eliminates the need for trusted central authorities, is the foundation of cryptocurrencies. However, some BC technologies go beyond that and provide smart contracts—the truly revolutionizing feature of the BC, which is not present in traditional web, cloud, and mashup architectures. Smart contracts constitute on-chain business logic that is executed within the blockchain network. Such execution can be verified by any network participant and thus trusted in the same way that any other transaction in a BC network is.

Smart contract code is written in a corresponding programming language (e.g., in Solidity for Ethereum, in Go or Java for HLF, in C#, Java or Python for NEO); it is then compiled to the bitcode suitable for a particular BC, and deployed to the network.

Once deployed in the BC network, a smart contract is addressed by its unique address, similarly to regular BC accounts. A smart contract highlights functions that can be used by other blockchain accounts. These functions represent a kind of an on-chain API for other BC accounts, and are accessible via the blockchain. A smart contract receives transactions addressed to it, with parameters required by a specific SC function embedded in the transaction. The smart contract processes the incoming request according to its programming logic and optionally launches events. The events can be later captured by other clients in the blockchain. The events can thus trigger those actions in IoT applications that rely on the blockchain and have to react upon changes to chain and smart contracts. 

### 3.2. IoT Applications with BC Support

Web, mobile, or embedded applications combine regular application logic (e.g., for user interfaces, sensor data acquisition, local data processing) with BC capabilities. Such capabilities can include a simple transaction exchange in the BC network or communications with an on-chain application part, i.e., a smart contract. The IoT applications use the BC via BC client API libraries and the BC client APIs that are exposed by the BC clients. These functional blocks of the IoT application part are described in more detail in the continuation of this section.

An unmanned embedded IoT system operates without direct user interventions, so a browser is not the appropriate environment for application execution. In that case an application is usually executed in some server side runtime environment (e.g., NodeJS for JS) and the appropriate BC client API libraries must be imported to the environment for proper operation. This is the foundation of an IoT device with BC support. 

There are two key modes of operation for BC-enabled IoT devices to work with and react upon the changes in the BC:

- In the first case a device is identified by a BC address/account. The BC transactions can be sent to and from this address. For the outgoing transactions to be properly signed by the issuers, the location of and secure access to the account key store are needed (see Section 3.3 for details). In this mode the device/application can, e.g., autonomously record its status in the chain.
- In the second case, an IoT device does not have its own BC account. However, even without it, a device can intercept the transactions or the events created by the smart contracts and the BC network. In this way the application can execute certain actions (e.g., toggle on a relay), if a corresponding transaction or event was recorded in the chain (e.g., transaction of some value to a specified BC address). This mode of operation is passive, as IoT devices/application cannot create transactions (operates as a sniffer), but it is much simpler in terms of secure key store management.

While rather distinct in their scopes, both modes of operation have practical value for IoT applications with blockchain support. In smart grids, for example, a passive sniffer could be used for a prepaid energy meter. The meter would intercept its expected status from the blockchain and provide electricity only if enough funds were available. If the consumption exceeds the prepaid quantity, the meter switches off the power. To do this, the meter does not create a transaction and does not need to have its own BC address. An active blockchain node would be required for metering where the device reports its status to the BC network. A meter reading would be reported through a transaction created in the meter and identified by that meters’ unique BC address. Any other more complex scenario (e.g., device automation, autonomous negotiations via the BC) requires active nodes, too. 

### 3.3. Building Blocks of an IoT Application for the Ethereum Blockchain

There are five key functional blocks present in an IoT application, with blockchain support to provide the desired functionality and communicate properly with the BC:

- The BC client is responsible for running the BC protocols and thus all communication with the BC network.
  This includes the management of blocks (keeping the local chain up-to-date) and transactions (e.g., sending outgoing transactions), listening to events, management of peers and the network, monitoring of chain status, managing the accounts or mining blocks. There are several Ethereum BC client implementations available, but geth [45] usually serves as the reference, because it is being developed by Ethereum Foundation developers. A popular alternative is the parity client [46].
  There are several synchronization options for the BC client, which affect communication, processing, and storage requirements for the device. Full syncing implies download, verification, and processing of all the chain blocks. In the initial stage fast syncing [28] downloads the transaction receipts along the blocks, and pulls an entire recent state database. Only when the chain reaches a recent enough state, fast sync switches to block processing. This results in much faster synchronization and less download traffic in the initial phase, but potentially opens additional security considerations. With the light syncing option the client only gets the current state. To verify elements, it needs to make inquiries to full nodes. The requirements for light syncing are reduced even further.
- The key store is the location of the private keys associated with a blockchain account. The keys are needed to duly sign the outgoing transactions and thus also access the funds in the account. A lost or stolen key store usually results in severe security breaches.
- The BC client API is a part of the BC client that exposes the clients’ capabilities.
  Through this client API the entire functionality of the BC client can be exploited. The API can be accessed through common programing and communication interfaces, usually the inter-process communication (IPC), HTTP POST, or Websocket (WS).
  The IPC can be applied if the application and the BC client run on the same physical device (local communication). The HTTP and WS on the other hand enable also a remote access to the BC client. The data passing through one of these channels is usually structured as JSON.
- BC client API libraries facilitate the application development and use of the BC client API.
  There are various implementations of these libraries available, for different programming languages and by different developers. In Ethereum such a library is the web3.js [47] (current version 1.0.0) for JavaScript programming. Other implementations may vary in their maturity. These programming libraries are included in the application projects.
  Apart from interfacing the BC client API, these libraries can provide additional features, as for example a local key store, which keeps and secures access to user accounts and keys, and facilitates the signing of outgoing transactions. This is of utmost importance for the IoT BC application development, as now the application code can manage the accounts easily, securely, and without user interaction.
- The application implements the desired functionality and utilizes the BC through the BC client API libraries.

The application programming code is, in the case of Ethereum, mostly written in JavaScript. The reasons for this are twofold. First, in both cases the JS BC client API libraries are the most advanced and proven, and second, it is suitable for browser-based applications, as well as the IoT device applications. 

### 3.4. Ethereum Transaction Lifecycle

Ethereum transactions transfer value between accounts, pass data to SC function calls, and deploy new smart contracts to the BC network. Once submitted to the BC network, the transactions are organized in blocks by mining nodes, which then execute consensus algorithms upon these blocks. A successfully mined block is added to the chain and the incorporated transactions become part of the irrevocable ledger of past transactions. The Ethereum BC client is responsible for the creation and submission of the transaction to the BC network. An Ethereum BC transaction that is compliant with the Ethereum BC protocol is a set of data that are serialized in the Recursive Length Prefix (RLP) format. In this format there are no parameter names and the input values are in hexadecimal format. Such a message is called a raw transaction. 

The BC client API libraries provide functions for signing the transactions and passing them on to the BC network. In the programming language of the application a transaction is presented as a data structure (object), which is then passed on to the corresponding functions. This data structure is unsigned and has no signature filed. In JS programing with the web3.js 1.0.0 library for example, the function sendTransaction() receives such a structure in JSON format, creates the appropriate signature, encodes the results in the RLP format to build the raw transaction, and broadcasts it to the BC network peers. The signTransaction() on the other hand only creates a raw transaction that can be then later passed to the network by, e.g., sendSignedTransaction(). For a transaction to be signed, the sendTransaction() and signTransaction() require access to an unlocked Ethereum account.

### 3.5. Stand-Alone Blockchain IoT Device Architecture

**Figure 2:**
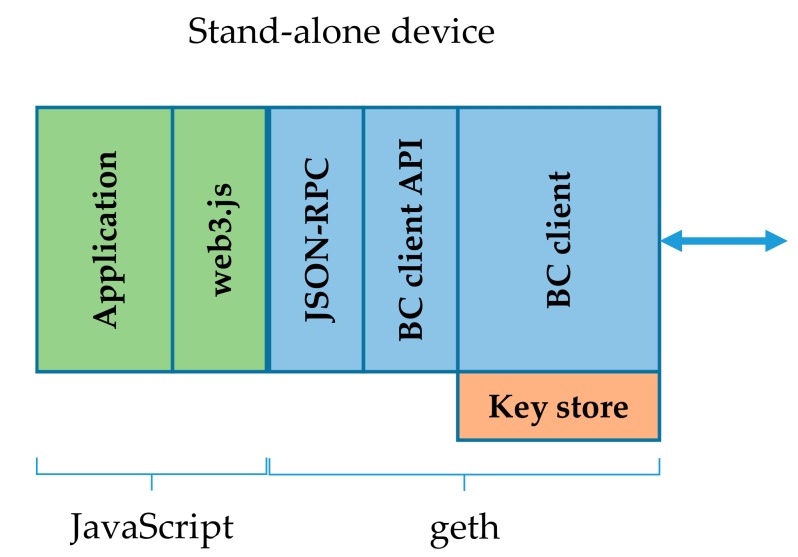
Stand-alone blockchain IoT device architecture [36].

The stand-alone blockchain device architecture is the initial architecture for the deployment of an end-system (including IoT devices) running applications with blockchain support. The BC client can be configured for full, fast, or light syncing. With full and fast syncing this architecture is only applicable in constraint-less devices and serves as the reference point for elaboration and investigation of practically feasible architectures, which are presented in Section 4. In the stand-alone device architecture, which is depicted in Figure 2, all the functional blocks run on the same physical device. As the BC client (geth) is running there as well, it imposes high demands on the CPU, memory, and storage. If the full BC synchronization is enabled, more than 225 GB [29] of Ethereum chain data must be counted in order to be transferred to and stored at the device. With fast synchronization the size of stored chain data is lower than in full chain, but is still about 130 GB. However, once the client is partially synced, additional growth of the chain data and the related communication traffic are the same in both cases. With light synchronization, communication traffic is further reduced to about 1.1 GB. This setup still requires a reliable and permanent communication channel. The key disadvantage of light syncing is unreliable smart contract event filtering. 

The key store in this case is placed locally and is unlocked by the geth upon the BC client initialization. The key risk in this architecture is the hardware security (stolen keys, if the physical device’s privacy is violated). 

The constraints from the perspective of a transaction management are summarized in Table 2. Our experience with such a setup showed that it is suitable only for the most powerful (IoT) devices. We tried to run the full client on a Raspberry Pi 3 Model B embedded system with a wired Internet connection. The syncing of the chain proved to be highly unreliable. We experienced unusually long synchronizations (syncing running for several days but still not completing), unexpected interruptions in synchronization, etc. While conducting these tests we had a reference client running on a regular computer (same IP network capacities) and syncing there was unproblematic. It is important to know that an unsynchronized BC prevents the application part from using any BC services. We tried running the geth in light mode, too. The syncing was more successful; however, we experienced severe problems in filtering the events that were launched by our smart contract. Some events were lost due to incomplete data information having been provided, despite the corresponding transactions being duly recorded and chain synced.

## 4. Ethereum Application Deployment Options for Constrained Devices 

Architectures of IoT applications with blockchain support heavily depend on the capabilities and limitations of the IoT devices where the applications are deployed. The IoT devices demonstrate a wide range of communication (bitrate, persistence of connectivity) and computation (CPU, storage) capabilities, ranging from dumb sensor nodes to fully equipped computers. It is therefore necessary to know these capabilities in advance, to properly select where particular functional blocks (Section 3.3) can run and how they are configured. The challenge is how to organize the required building blocks and implement devices that functionally resemble the stand-alone IoT node—presented in Section 3.5—but with architectures that address possible constraints. All the considerations about the architecture and configurations aimed at providing a reliable execution of the IoT application logic and of the workflow for the Ethereum transactions (creation, signing, submitting, monitoring) and events. 

Starting from the stand-alone IoT node architecture, there are two possible architectural choices at disposal:

- the location of the (full) blockchain client, and
- the location of the key store

The first step is to move the blockchain client out of the IoT device and run it on a constraint less network proxy. We call this architecture the remote geth client architecture. Further on we have two possibilities for the key store location. The key store can remain at the IoT device, referred to as a remote geth client with local key store architecture. The second step is to also place the key store to the remote location and keep it with the blockchain client. We refer to this as the remote geth client with remote key store architecture. 

Remote geth client architecture to some extent digresses from the fully decentralized peer-to-peer philosophy that is fundamental to the distributed ledgers and BCs. It requires a certain level of trust in the proxy node where the remote client is running. On the other hand, similar approaches are taken, e.g., for most mobile BC clients (that mobile app has to trust the server that provides it the BC functionality). The recent fog computing developments and 4/5G network architectures also indicate that network edge nodes could serve as application gateways, providing functionality to the end nodes. In deployment of decentralized applications we can rely on providers like Infura [48], too. Instead of running Ethereum nodes on our own, a remote node with the API and a reliable BC network connectivity can be provided as a service. In a similar way some other blockchain APIs are made available [49]. As part of the BigQuery Public Datasets program, in 2018 Google Cloud released datasets consisting of the blockchain transaction history for Bitcoin and Ethereum and introduced additional cryptocurrencies later [50]. As the objective of BigQuery is different, this data is meant for BC network and smart contract analytics and not for the deployment of decentralized applications.

However, in addition to the favorable impact on computation, storage, and communication requirements, architectural variations may affect the security of the overall decentralized application. The security impact caused by the architectural variations cannot be completely avoided, but understanding the possible security implications makes the security risks predictable and the requirements acceptable. In particular, the hardware security risk can increase and the level of decentralization can decrease. The possible security implications are therefore also considered in selecting the architectural variations. The stand-alone node clearly provides the highest level of decentralization and the related trust, which is the blockchain’s key security attribute. Transferring the blockchain functionalities from the end-device to remote nodes requires trust in these nodes. The level of the required trust varies according to the blockchain services that these nodes are providing. The architectural options in the IoT device design for blockchain range from the (practically infeasible) full nodes, to web-hosted exchanges, where the entire end device utilizes none of the blockchain functions directly. There is less risk, e.g., if a device creates and signs its own transactions and the remote node only distributes them to the blockchain. If the key store is kept at the remote node and the transactions are signed there, then the level of trust in the node must be high. If remote nodes are applied as a service, no application key stores can be kept at the server for security reasons. 

The deployment options are compared in Table 3 and analyzed following subsections. 

The remote geth client architecture can be practically deployed in, e.g., smart grid systems. For example, if the power line communications (PLC) is applied for connectivity, the blockchain proxy could reside at a message-aggregating gateway or data concentrator, which is already located at the secondary substation and terminates the PLC connections. A secondary substation usually has its own local area network and broadband connectivity towards higher levels of the grid architecture, so running a full client there is possible. 

### 4.1. Remote Geth Client with Remote Key Store

**Figure 3:**
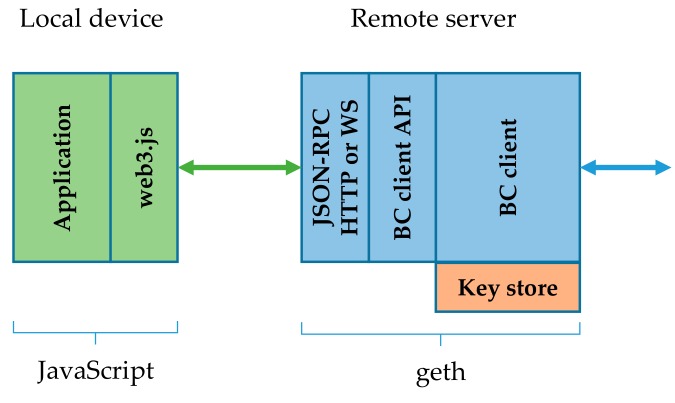
Remote geth client with remote key store [36].

With remote geth clients and remote key stores we run geth on a separate, constraint-free server. The JavaScript application of course remains at the local IoT device. The most resource intensive part is thus moved from the IoT device. A remote server exposes the geth functionality over JSON-RPC API, with HTTP or WS as the transport channel. In this setup the key store remains at the server. The key store is applied at the geth client initialization, just as in the case of a stand-alone node. The remote geth client with remote key store architecture is depicted in Figure 3.

This architecture actually proved to have a practical value. A local device was successful in running the application part, while a remote server seamlessly ran the geth. This approach most efficiently reduces computation and storage requirements set to the local device. It also importantly reduces the communication requirements, because only the incoming notifications about subscribed events and outgoing transactions have to be transferred. With the remote geth client architecture the blockchain synchronization data does not access the local device. As presented later in Section 5, a typical transaction submitted by the application to the geth in the form of JSON-RPC over HTTP was comprised of one HTTP POST request and a corresponding response. In this request the JSON transaction object is passed to the BC client API function call. The size of the request messages was about 800B. The response message was smaller at 280 B. When WS was used instead of HTTP, the messages were roughly 200 B smaller. This does not seem like much, but it can still exceed the communication limits of low bit-rate devices. This is especially true if not merely a limited number of transactions is passed over HTTP/WS, but also some event filtering from web3.js is applied that utilizes the polling principle, generating a constant network load. 

However, this architecture has potential security risks we need to understand. If geth is run with the key store unlocked, then anyone accessing the geth with JSON-RPC over HTTP or WS can create transactions signed with this key. There is no access control to HTTP or WS built into the geth, so we need to plan the IP network layer’s security very carefully in this case. These risks are relevant only where the IoT device acts as an active transaction creator. If it runs in passive mode (sniffing the chain for transactions and events), then no key store is required, so there are no risks in this respect.

Smart grids are viewed as a possible application of this architecture, predominantly for devices running in passive mode, as for example load control via blockchain or device management. 

### 4.2. Remote Geth Client with Local Key Store

**Figure 4:**
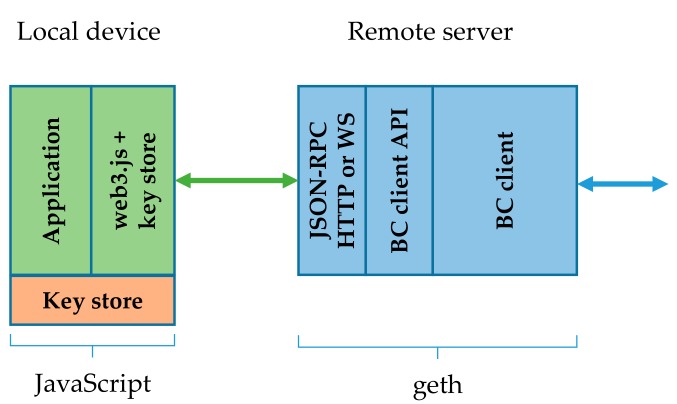
Remote geth client with local key store.

The remote geth client with local key store architecture—which is depicted in Figure 4—and the one with remote key store differ in how the key store is positioned. As this is no longer placed on the server, the security risk of sharing the same geth client among several devices diminishes. In fact, in terms of security, only the client’s availability remains a relevant issue. The blockchain data kept by the client is readily available in the BC network to any other participant and is not meant to be private. In this case, however, the application has to (create and) submit raw transactions, including the signature and apply proper serialization. Key store management and signing of transactions is already supported in the web3.js. 

Due to the remote location of the client this approach also efficiently reduces computation and storage requirements set to the local device. The slightly increased computational requirements are related to the signing of transactions, now done locally. It also importantly reduces the communication requirements, just like the architecture with the remote key store. The remaining traffic volumes are comparable to the ones in the remote key store approach, as discussed in Section 4.1. Interestingly, passing raw transactions instead of JSON transaction objects over the HTTP/WS did not result in smaller message sizes, which were expected due to the more efficient RLP encoding. The raw transaction namely includes the signature and transaction hash (not present in JSON transaction object), resulting in messages that in this particular case were about 40B larger.

In terms of security this architecture is much closer to the initial idea of a fully decentralized blockchain network. Every local device is identified by its own Ethereum account and keeps its key store. The need to trust the network proxy is largely reduced and is concentrated to the availability of proxy services. Even a selection of arbitrary geth proxies is possible. However, additional hardware security risks emerge, due to local positioning of the key store and possible device tampering.

Nonetheless, the hardware security requirements are at least to some extent readily addressed in the current (non blockchain) IoT devices, as for example smart meter solutions. Smart meter manufacturers should provide security levels in their devices that are comparable to the ones in online payment systems. This includes secure storage of cryptographic keys and certificates. For example, the study [51] addresses the need for cost-effective tamper-resistant smart energy devices, and [52] the security standards supporting smart grid reliable operation, including the role of trusted computing platforms for smart grid. Nevertheless, it is true that actual secure implementation is still a vendor-specific issue, affected by cost and resource constraints, and performance considerations [53].

### 4.3. Proprietary Local-Device to Remote-Server Communication

The remote client lets us successfully address the computational and storage constraints. Even though the communication load is drastically reduced compared to the full node architecture, it still remains too big for low-bit-rate networks. The communication between the local device and the geth client over the HTTP/WS was not optimized for minimum communication loads. The redundancy is for example in hexadecimal encodings of raw transactions in API calls, application layer overhead and headers, and event notification management. 

**Figure 5:**
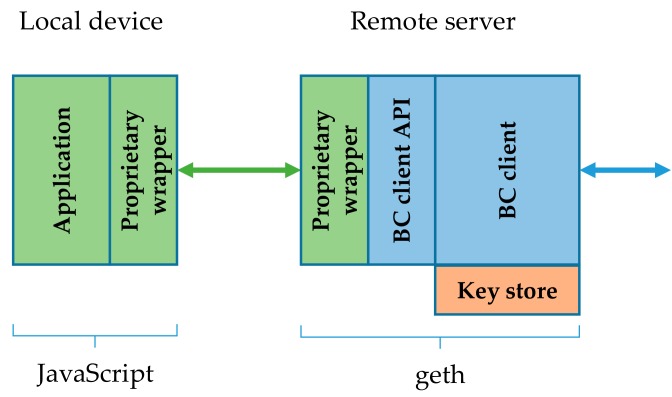
Proprietary wrapper for communication between the device and the remote server.

As the last option we therefore propose a proprietary communication protocol between the IoT local device end the remote (geth) server, as seen in Figure 5, discarding the existing JSON or RLP data formats. We see two benefits in this; first, the communication bandwidth requirements can be reduced to the minimum, and be thus able to communicate over low bit-rate channels, too. Second, we can apply advanced server access controls to minimize security risks described in the remote geth client with remote key store architecture. According to our knowledge no such solution has been provided for the Ethereum network.

The approach could be implemented as a proprietary communication wrapper, with corresponding instances placed at the proxy node and at the local device. At the time of writing the web3.js library only facilitates the remote key store approach. Local creation and signing of the transactions with web3.js is only possible if the library has a Websocket connection (which is discarded in this architecture) to a geth client. However, it has been already announced that, in the future, no connection to the geth will be required, so the local key store with proprietary protocols will become viable, too.

This approach can reduce the size of exchanged messages to only several bytes and at the same time does not increase the computation and storage requirements compared to the first two remote geth client architectures. The frequency of the messages can be reduced too. The wrapper at the server does not only have to be transparent, but it can also implement some of the event notification and management logic that is otherwise executed at the local device. Even verification, for example, can be done at the proxy, so only notifications of previously verified events are passed to the local device. Without a proprietary wrapper the device would have to verify the event by issuing additional messages to the proxy. 

## 5. Communication Traffic Measurements and Results

To be able to understand the full scope of possibilities and constraints of Ethereum-based decentralized applications, we had a prototype developed for an Ethereum BC-controlled IoT electric switch and smart meter, dubbed *Swether*, along with smart contracts and Ethereum-compliant web applications [34]. The application deployment options presented in this section and the communication traffic analysis were verified with this pilot DApp setup. We were running the system in the Ropsten public Ethereum test network. The traffic captures and analysis were made with Wireshark. We measured and analyzed the network traffic to the client and the traffic passed between the application and the geth client to get practical insight into possible communication constraints. 

### 5.1. Experimentation Setup and Scenarios

The *Swether* [34] is a prototype smart grid device that can act as an electric switch controlled through the Ethereum network and an energy meter that reports its metering to Ethereum. This requires both key modes of operation for BC-enabled IoT devices. If a device is identified by a BC address/account, the BC transactions can be sent to and from this address. In this case we were able to analyze the transaction-related traffic. If a device operates in passive mode, it only intercepts the event notifications created by the smart contracts or the BC network. In this case we were able to focus on the event notification-related traffic. In both modes there we expected a substantial share of periodic communication traffic needed for blockchain participation, not directly related to application-specific transactions or events. For experimentation purposes we configured a *Swether* in the various deployment options presented in Section 4.

In a typical user scenario a user would book a plug in a *Swether* device for a desired time period or energy quantity, and confirm the booking with a transaction to the Ethereum network sent from the BC-enabled web browser (see Section 3.2 for details). The smart contract would validate the request and launch the events to the blockchain. The events would be intercepted by the *Swether* device. If required, the *Swether* device would report consumption metering via a transaction sent to the smart contact. 

**Figure 6:**
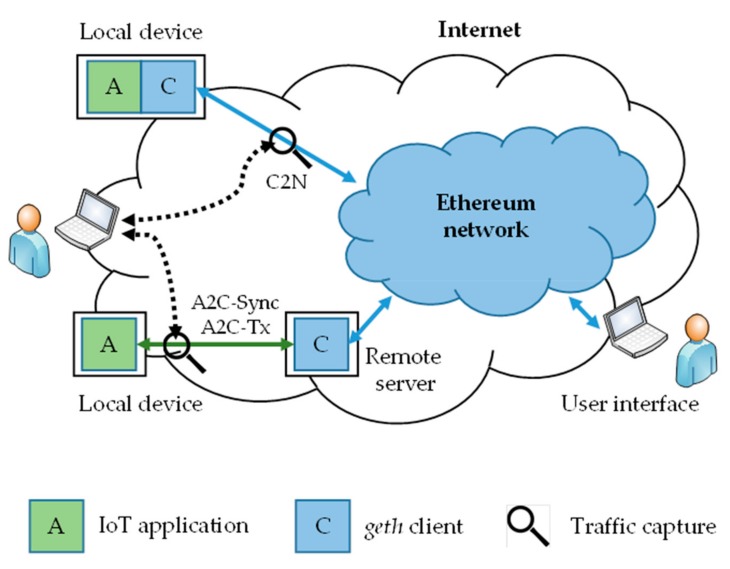
Pilot setup for traffic measurements [nist.sp.1108R3].

The prototype deployment architecture and traffic capturing points are presented in Figure 6. We can distinguish between three traffic categories in this topology, which are labeled in the figure and explained in the continuation. 

For stand-alone end-devices, both the IoT application and the geth client run locally on the IoT device, so the captured traffic includes the entirety of the device-to-Ethereum communication. The application and the client in this case communicate internally, over the IPC. The traffic between the geth client and the Ethereum network (labeled as C2N) is predominantly caused by the periodic blockchain synchronization, but also includes peer-discovery communications, transaction broadcasting, and the like. As application developers, we have little impact on this traffic since it is operated by the geth. An alternative is developing proprietary BC clients, but with that we lose the benefits of any public Ethereum networks. 

With remote servers the geth client is moved from the local device and the traffic refers to the JSON-RPC via HTTP or WS (or a proprietary protocol, if implemented) between the device and the remote server. The second traffic category is the periodic traffic between the application and the remote geth client (labeled as A2C-Sync). It results from the client updating the application about the chain status. This is not the chain data as in C2N-Sync, but merely the notifications about new chain block arrivals, so that the application keeps in contact with the client. The third category is traffic related (labeled as A2C-Tx) to the exchange of transactions and event notification between the application and the geth. This traffic is not periodic and is entirely application-specific. The amounts of A2C-Sync and A2C-Tx do not depend on the geth client-syncing mode (full, fast, and light).

### 5.2. Measurement Results

Table 4 summarizes the results of periodic communication traffic measurement (C2N and A2C‑Sync) between the device and the remote geth client.

Table 5 summarizes sizes of transactions to a smart contract address passed from the device to the client.

## 6. Discussion

### 6.1. C2N: Traffic between the Geth Client and the Ethereum

In full client architecture with full and fast syncing, the traffic to the geth client from the Ethereum network was measured over a period of about 60 minutes. Already before the capture the client was fully synchronized (i.e., the entire blockchain stored at the client). We disabled any application-related events. The traffic therefore resulted only from the regular synchronization of new blocks. The average data rate was about 18 kB/s and about 10 kB/s in download only. This is a traffic volume that is directly determined by the blockchain network, protocol, and client, and we do not have much influence on it. Even without any additional application-related data, this traffic to the client is needed to keep the local copy of the chain in synchronization.

The light syncing option proved to be inappropriate for our decentralized applications. Despite that, for the reference we measured analyzed the traffic to the geth client with light option. The average data rate was about 1–2 kB/s

### 6.2. A2C-Sync: Periodic Traffic between the Application and the Geth Client

In a remote client architecture the traffic volume between the node and the full client depends on the applied communication protocol (HTTP or WS), the amount of the data passed to a smart contract in a transaction, and event frequency. Some of the events that result in an exchange of packets are periodic, as for example the indication passed from the client to the node about a new block in the chain. For HTTP this traffic volume was about 2400 B/s and for WS it was 170 B/s. This vast difference results mainly from different modes of operation and not from the applied communication protocol. Less efficient polling is applied for HTTP, and, with WS, the geth client pushes the event indication to the application only when a new block appears.

### 6.3. A2C-Tx: Transaction and Event Notification-Related Traffic Between the Application and the Geth Client

The appearance of other events is not periodic and results from the DApp’s characteristics and use. In our use case each new booking of a charging plug resulted in one captured event and a corresponding confirmation. Besides, the application required one additional event verification after a predetermined number of new blocks in the chain for security reasons. The event capture notification and the corresponding verification resulted in about 2000 B of traffic. 

Further, we analyzed the traffic volumes to pass a transaction from the node to the client. A charger would create such a transaction, e.g., to notify the smart contract about the actual amount of energy consumed during a single charging. We considered two cases; with local key store a transaction is created and signed at the node and serialized in the RLP format. Such raw transactions (Raw Tx) are passed by the client to the Ethereum network. For remote key store the node passes a JSON transaction object to the client, which then signs, serializes, and broadcasts it to the network. The sizes of packets for both cases are shown in Table 5. Both the HTTP and WS communication protocols were applied. There is the web3.js function provided in the table to indicate how the transaction was built. 

The sizes given in Table 5 refer to the transactions that include some data for the smart contract. The minimum size of a raw transaction (no communication headers included) with no additional data was 104 B. However, when a raw transaction is submitted to the client, it is converted into a hexadecimal string, which is the actual parameter in the client API function call. The string encoding duplicates the size of the raw transaction in bytes to 208 B. Additional data for the SC function call can be included in the transaction. In our case the size of this data was 278 B and 268 B. Beside these fields to build the required JSON, input for the API function is also added. We can estimate that the entire request frame is about 300–500 B if there is no SC data. The SC data size is added up to the frame size with no data. Request frame sizes in HTTP are about 200 B bigger than in WS due to more compact application layer headers. The size of the frame with a JSON transaction object is 45 B smaller than a corresponding raw transaction. While the raw transaction was expected to be serialized more efficiently due to RLP, that was not the case. The increased size of frame with a raw transaction results from the additional RLP to string encoding and from the signature, which is added to the raw transaction (but is not a part of the JSON Tx object). 

## 7. Conclusions

Blockchain technologies are attracting immense attention, both positive and negative, mostly due to current hectic activities in cryptocurrency markets and ICOs. Our research is focused on the technical aspects of blockchain technologies. In our view, which is backed by the initial research, developments, and application examples, there is a vast IoT application opportunity for these technologies, especially in relation to smart grids and smart energies. Our research provides directions for IoT application designers to enable them to select the appropriate system design and avoiding unrealistic expectations imposed to the IoT devices and BC technologies. The architectural approach can be thus shaped according to the intended use and the specifics of the planned IoT system. 

We investigated how to match the requirements and constraints of the IoT devices found, e.g., in the smart grid customer domain, as for example smart meters, smart grid gateways, or data concentrators. In standalone device architecture, the application and the blockchain client run on the same device. This imposes computational, storage, and communication requirements that smart meters or gateways cannot meet, and makes the implementation of blockchain nodes for smart energy as standalone end-devices impractical or even impossible. We measured and analyzed the communication traffic of a standalone blockchain node and compared it to the traffic where the blockchain client was moved to a remote server. We found out that it is possible to distinguish between two traffic categories. Periodic communication traffic is needed for blockchain participation, i.e., syncing the blockchain or receiving event notifications about synchronization. Traffic that results from event notifications and transaction exchange is not periodic and depends on application operation. For application operation we therefore analyzed the sizes of particular transactions instead of the average load. The periodic traffic of a standalone Ethereum node is about 18 kB/s. 

The most resource-demanding part in the remote server architecture—i.e., the geth client—is moved from the end-device and placed on a server. This significantly reduces the computation and storage requirements, which can now be met by, e.g., current smart meters. The periodic traffic of a device is strongly reduced from the initial 18 kB/s, too. For HTTP it is about 2400 B/s and with Websocket about 170 B/s. The difference in the two is mostly due to inefficient polling applied by the geth client in the case of HTTP. 

A notification about a captured event and the corresponding verification resulted in an exchange of about 2000 B. A transaction sent from the application on the device to a remote client resulted in a message of about 500 B with HTTP and in 300 B with Websocket, due to the more compact application layer headers. If there are additional smart contract input data in the transaction, they are added to the values above. The size of smart contract data is entirely application-dependent. The key store location, which affects the serialization of the transaction, only had a small influence on the transaction-related data. With raw transactions the messages were 45 B larger that when passing the JSON transaction objects. The reason for this is the transaction signature, which is included in the raw transaction. This is a positive finding, since the placement of the key store can be now selected predominantly based on the security requirements and not to meet the communications constraints. 

To glean additional insight into our results, we mapped our measurements to estimated NB-IoT and LoRaWAN client conditions. For NB-IoT with a peak rate 250 kb/s and 100 connected end devices, the available bitrate for one end device results in an average rate of 2.5 kb/s (or 312 B/s). This means that the node could be kept synchronized if WS and remote client architecture were applied. The periodic traffic would use 55% of the available bitrate. A remote node with HTTP and a full node would exceed the available bitrate in this case. If all the remaining bitrate (12 MB/day) were dedicated to blockchain transactions, this would additionally allow for transfer of about 40,000 transactions. LoRaWAN is not appropriate for periodic traffic, because of impermanent connectivity and excessive traffic even for WS. It could be suitable to transfer transactions and event notifications. In LoRaWAN connectivity, 100 connected clients and 1% duty cycle result in 70 seconds of air time per device per day. This is about 800 kb/day or 100 kB/day per device. This means that about 333 transactions or 50 event notifications per day could be transferred for the device.

Remote server architecture helps reduce the communication constraints, but the traffic still exceeds the capacities of narrow-band low-bit rate networks. This indicated the direction of future expansion of our research. We are therefore currently specifying and implementing a low bit-rate Ethereum (LBE) protocol for communication from proprietary device to remote client, with the ambition to test it in LoRaWAN and other low bitrate networks. We expect to further reduce periodic traffic volumes, so as to manage the number and limit the size of event notifications, and reduce transaction object sizes. We also plan to set up blockchain nodes for the Ethereum, Bitcoin, IOTA, and NEO networks to continuously monitor the actual system requirements and make these findings available to the research community and thus help to overcome the vaguely defined system requirements for blockchain devices.

## Figures and Tables

**Table 1:** Expectation put on blockchain technologies in the Internet of things (IoT).

Expectations	Facts	Remedies
Build trust	Yes, trusted transaction ledgers based on decentralized trustless infrastructure.	-
Scalability	Very limited in major public BC networks.	New consensus algorithms; state channels; non-blockchain based distributed ledger technologies; private or permissioned networks, cross-chain solutions
Accelerated transactions	Very poor in major public BC networks, sometimes long transaction confirmation delays and low throughput.	Protocol improvements (state channels, sharding); private or permissioned networks; new BC principles (tangle)
Data monetization, micro payments (cost reduction)	Far from being true in major public BC networks. Transaction costs in the Ethereum are about 0.1 USD and in Bitcoin about 2 USD.	New consensus algorithms; private or permissioned networks
Device autonomy and M2M transactions	Yes, considering the previously mentioned limitations.	-

**Table 2:** Possible constraints in an Ethereum transaction management.

Lifecycle Point	Possible Constraints
Creation of a transaction object	No additional constraints
Keeping the key store	Hardware security
Signing a transaction	Computational constraints – might be an issue for very simple devices
Serialization	No constraints
Submitting to the BC network	Communication constraints – if low rate communications are applied
Adding transaction to a chain block	Not relevant for end devices
Syncing the full node, including full and fast mode	Communication and storage constraints - to demanding for an end device
Syncing the light node	Communication constraints – permanent communication channel is required; Application constraints – unreliable smart contract event filtering
Informing about the status of the chain from a remote full client	Communication constraints - if low rate communications are applied

**Table 3:** Comparison of deployment options.

Requirement/Feature	Standalone Full Node	Remote Client and Remote Key Store	Remote Client and Local Key Store	Remote Client and Proprietary Protocol
Computation	High	Low	Moderate	Low–Moderate ^1^
Communication	High	Moderate	Moderate	Low
Storage	High	Low	Low	Low
Decentralization	Highest	Moderate	High	Moderate–High ^1^
Device security risk	High	Low	High	Low or High ^1^

^1^ A range of options is possible.

**Table 4:** Periodic communication traffic between the device and the geth client.

Architecture	Label	Periodic Traffic
Stand-alone device – full/fast	C2N	18 kB/s
Stand-alone device – light	C2N	1-2 kB/s
Remote geth client with HTTP	A2C-Sync with HTTP	2.4 kB/s
Remote geth client with Websocket	A2C-Sync with Websocket	0.17 kB/s

**Table 5:** Sizes of transactions to a smart contract address passed from the device to the client.

Communication ->	HTTP		Websocket	
Tx Serialization ->	Raw Tx ^1^	Tx Object ^2^	Raw Tx ^1^	Tx Object ^2^
SC data [B]	278	268	278	268
Tx –no SC data [B]	208^3^	178	208^3^	178
Tx -with SC data [B]	486	446	486	446
Request payload [B]	556	511	556	511
Request frame [B]	805	760	618	573
Response payload [B]	103	103	103	103
Response frame [B]	280	280	159	159
web3.js function	sendRawTransaction()	sendTransaction()	sendRawTransaction()	sendTransaction()

^1^ Signed transaction passed from the device to the client in RLP serialized form. ^2^ Transaction object passed from the device to the client in JSON format. ^3^ Raw transaction encoded as a hexadecimal string.
